# Body weight prediction using different data mining algorithms in Thalli sheep: A comparative study

**DOI:** 10.14202/vetworld.2021.2332-2338

**Published:** 2021-09-06

**Authors:** Ansar Abbas, Muhammad Aman Ullah, Abdul Waheed

**Affiliations:** 1 Department of Statistics, Government Degree College for Boys, Makhdoom Rasheed, Multan, Pakistan; 2 Department of Statistics, Bahauddin Zakariya University, Multan, Pakistan; 3 Department of Livestock and Poultry, Bahauddin Zakariya University, Multan, Pakistan.

**Keywords:** artificial neural network, body weight, classification and regression tree, Chi-square automatic interaction detector, exhaustive Chi-square automatic interaction detector, Thalli sheep

## Abstract

**Background and Aim:**

The Thalli sheep are the main breed of sheep in Pakistan, and an effective method to predict their body weight (BW) using linear body measurements has not yet been determined. Therefore, this study aims to establish an algorithm with the best predictive capability, among the Chi-square automatic interaction detector (CHAID), exhaustive CHAID, artificial neural network, and classification and regression tree (CART) algorithms, in live BW prediction using selected body measurements in female Pakistani Thalli sheep.

**Materials and Methods:**

A total of 152 BW records, including nine continuous predictors (wither height, body length [BL], head length, rump length, tail length, head width, rump width, heart girth [HG], and barrel depth), were utilized. The coefficient of determination (R^2^), standard deviation ratio, root-mean-square error (RMSE), etc., were calculated for each algorithm.

**Results:**

The R^2^ (%) values ranged from 49.28 (CART) to 64.48 (CHAID). The lowest RMSE was found for CHAID (2.61), and the highest one for CART (3.12). The most significant predictors were the HG of live BW for all algorithms. The heaviest average BW (41.12 kg) was observed in the subgroup of those having a BL of >73.91 cm (Adjusted p=0.045).

**Conclusion:**

Among the algorithms, CHAID provided the most appropriate predictive capability in the prediction of live BW for female Thalli sheep. In general, the applied algorithms accurately predicted the BW of Thalli sheep, which can be very helpful in deciding on the standards, available drug doses, and required feed amount for animals.

## Introduction

The live body weight (BW) of sheep at different ages of their lifecycle is a significant trait for judging their adaptive performance. Knowing the live BW of small ruminants is important for breeding, proper feeding, and maintaining a healthy physiological condition. The BW is supplemented with measurements that describe an individual or population more absolutely than the conventional methods of weighing or grading. It gives sufficient information on the ­morphological structure of the animal as well as its physiological condition [1]. Furthermore, the body measurements of the animals are essential for establishing breed standards [2].

In the literature, various reports found a great figure for the estimation of live BW using the main predictors, such as morphological and testicular measurements in different sheep and goat breeds. Different researchers used various statistical techniques, that is, correlation analysis [3], principal component analysis in multiple linear regressions [4], and simple and multiple linear regressions [5-9] for live BW prediction. In terms of the classical assumptions, the Chi-square automatic interaction detector (CHAID), exhaustive CHAID, classification and regression tree (CART), and artificial neural network (ANN) types, such as the radial basis function (RBF) and multilayer perceptron with one (MLP1) and two (MLP2) hidden layers, have recently been used to perfectly indicate the body measurements in relation to live BW in sheep and goat breeding studies [10-12]. As the above-mentioned advantages have been considered in sheep breeding, data mining algorithms for live BW prediction using selected body measurements have been investigated. Yakubu [13] preferred the CART algorithm for the BW prediction of Uda rams. Mohammad *et al*. [14] predicted the BW of Balochi sheep using the exhaustive CHAID algorithm. A study by Ali *et al*. [10] predicted the live BW of Harnai sheep using the CART, CHAID, exhaustive CHAID, and ANN algorithms. In the prediction of lactation milk yield, Karadas *et al*. [15] tested the predictive capabilities of the CART, MLP, CHAID, and exhaustive CHAID algorithms. Another study by Eyduran *et al*. [16] also predicted the BW of Pakistani Beetal goat on the basis of six different traits, that is, head girth, neck length, diagonal body length (BL), belly sprung, shank circumference, and rump height using the CART, CHAID, ANN, and MLR algorithms.

In Pakistan, despite earlier research on the live BW prediction using different data mining algorithms in different sheep (Harnai, Balochi) and goat breeds [10,12,14,16], the live BW of the Thalli sheep in Southern Punjab, Pakistan, has not been fully investigated. Therefore, this study aims to determine the best data mining algorithm with respect to its predictive performance among the CHAID, exhaustive CHAID, CART, and ANN algorithms in the prediction of live BW using selected body measurements in female Pakistani Thalli sheep.

## Materials and Methods

### Ethical approval

Ethical approval was not required for this study as different body measurements were collected from sheep and each measurement was taken according to their operational procedures.

### Study period and location

This study was conducted from March 2018 to June 2019. The sheep were sampled from two different government livestock experimental stations, namely, “Rakh Ghulaman” located in Bhakkar District and “Rakh Kheirewala” located in Layyah District in Punjab, Pakistan.

### Data collection

In the present study, the data of 152 female Thalli sheep at varying ages ranging from 30 to 48 months were used. The sheep were sampled from two different government livestock experimental stations, namely, “Rakh Ghulaman” located in Bhakkar District and “Rakh Kheirewala” located in Layyah District in Punjab, Pakistan. Random sampling was used for sheep selection. All healthy sheep that did not receive any medication and had no physical disability were included in the study. A self-administered questionnaire was used to obtain information regarding age and morphological measurements. The morphometric traits, that is, BW, wither height (WH), BL, head length (HL), rump length (RL), tail width, head width (HW), rump width (RW), heart girth (HG), and barrel depth (BD), were measured on each sheep. The body measurements were done using a tailor’s tape measure in centimeters (cm), whereas BW was recorded using a weighing machine in kilograms (kg). These measurements were taken (in centimeters) in a standing position according to the standard procedures. Data collection activity was made by the same person to avoid between-individual variation. Table-1 shows the descriptive analyses, i.e., mean, standard deviation (SD), and percentage coefficient of variation (CV, %), of each quantitative variable.

**Table 1:** Descriptive statistics for body weight and body measurements of female Thalli sheep.

Body measurements	Mean	SD	CV (%)
Body weight (kg)	33.39	4.40	13.17
Withers height (cm)	71.87	4.16	5.78
Body length (cm)	72.11	4.84	6.71
Head length (cm)	28.37	2.21	7.78
Rump length (cm)	15.20	2.15	14.14
Tail length (cm)	12.69	3.26	25.68
Head width (cm)	10.62	1.02	9.60
Rump width (cm)	20.10	3.62	18.00
Heart girth (cm)	78.38	5.72	7.29
Barrel depth (cm)	47.62	3.79	7.95

SD: Standard deviation; CV: Coefficient of variation

### Statistical analysis

The first tree-based algorithm used for the prediction of the live BW of female Thalli sheep was CHAID. This algorithm is usually used to categorize the subsets of predictors that best depict the dependent variable. The basic objective of the CHAID technique is to minimize variation within the nodes to construct homogenous subgroups in the optimal regression tree diagram with significant predictors [17]. The second algorithm used for the prediction of the live BW of female Thalli sheep was the exhaustive CHAID algorithm. In the advancement of the CHAID algorithm, the exhaustive CHAID algorithm is based on three-stage-data mining algorithms (i.e., merging, partitioning, and stopping) that recursively use multiway splitting to form homogenous subsets on the basis of Bonferroni adjustment until the difference between the observed and estimated values in response variable is minimal [18,19]. Although the exhaustive CHAID algorithm has the same splitting and stopping rules as the CHAID algorithm, the merging step is more exhaustive than the CHAID algorithm, by continuing to merge categories of the predictor variable until only two super categories are left. Moreover, the exhaustive CHAID algorithm can find the best split for each predictor variable [20]. The third tree-based algorithm used in the present study was the CART algorithm [21]. It is a recursive splitting method and is used both for regression and classification problems. In the CART algorithm, the dependent variable is scale, whereas the independent variable can be scale or categorical. Moreover, it creates a binary split [22], and the best input variable is chosen by using a range of diversity procedures [15]. The CART algorithm creates more homogenous subgroups than the CHAID algorithm using pruning. By default, the maximum number of levels (tree depths) is five for CART and three for the CHAID algorithm. Tenfold cross-validation criteria were applied, and the minimum number of cases for parent and child node was set at 10:5 to correctly model the relationship between the response and independent variables as well as to get the best possible decision tree structure. Finally, ANN was used for live BW prediction in female Thalli sheep. ANN biologically resembles the human brain. It consists of three layers, that is., input, hidden, and output layers, and is used with one hidden layer on the source of MLP, which is also called a feed forward neural network to predict BW using the body measurements [10,23]. The data were at random and categorized as the training set (70%) and verification set (30%).

To compare the predictive capability of the CHAID, exhaustive CHAID, CART, and ANN algorithms in the tenfold cross-validation, the following goodness-of-fit criteria were used [11,12].

Coefficient of determination (%)


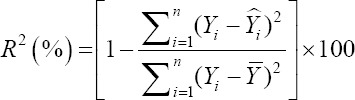


Adjusted coefficient of determination (%)


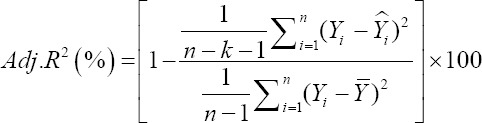


Standard deviation ratio (SD ratio)


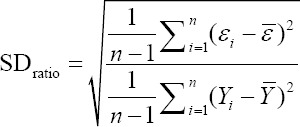


Root mean square error (RMSE)


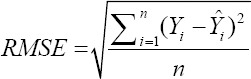


Coefficient of variation (%)


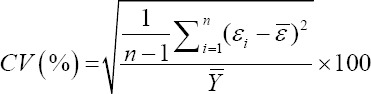


Mean absolute percentage error


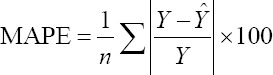


Relative approximation error (RAE)


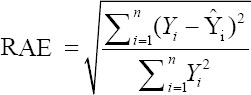


Akaike information criterion


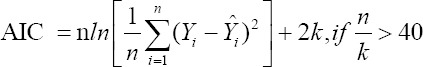


Or


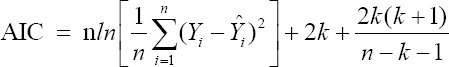


Where *Y_i_* and Ŷ_i_ are the actual and predicted BW values of the i^th^ sheep. ε*_i_* is the residual value of i^th^ sheep, −*Y* and −ε are the mean of actual BW and residual values. K is the number of input variables which are used in the model, and n is the total sample size. We also calculated the Pearson correlation co-efficient (r) between actual and predicted BW values. All of the above stated algorithms, that is, CHAID, Exhaustive CHAID, CART, and ANN are available in statistical software “Statistical Package for the Social Sciences (SPSS)” version 23.0 (SPSS Inc., Chicago, IL, USA) which were utilized for predicting BW of sheep on the basis of different morphological characteristics and a and p-value for splitting equal to 0.05. Moreover, the Bonferroni adjustment was utilized to correct for the p-values of the best predictor at each split in the CHAID algorithm.

## Results and Discussion

In the present study, first, we predicted the BW of 152 female Thalli sheep using multiple linear regressions. The BW prediction equation was *BM*=−32.5+0.19 WH+0.19 BL+0.21 HL+0.13 RL+0.05 TL−0.37 HW+0.07 RW+0.26 HG+0.19 BD along with R^2^=0.519, indicating that the 51.9% variation in the BW was explained by the predictors. Table-2 and Figure-1 show the results related to the performance of the CHAID, exhaustive CHAID, ANN, and CART algorithms to predict the BW. In the present study, we selected the best algorithm having the greatest r, R^2^ (%), and adj-R^2^ (%) values but the lowest SD ratio, RMSE, MAPE (%), RAE, CV (%), and AIC values. The mean BW prediction on the basis of the ten-fold cross-validation procedure showed that the CHAID algorithm was more superior to the exhaustive CHAID, ANN, and CART algorithms (Table-2). In line with our results, some earlier reports highlighted the biological advantage of the CHAID algorithm in BW prediction [11,14,22]. The Pearson correlation coefficient (r) between the estimated and observed values of BW was significantly higher (p<0.05) for the CHAID algorithm than for the exhaustive CHAID, ANN, and CART (i.e., r=0.80>0.75, 0.71, 0.70). Similarly, some other model quality criteria, such as R^2^ (%) and Adj-R^2^ (%), were also higher (i.e., R^2^ (%)=64.48>57.15, 51.69, 49.28; Adj-R^2^ (%)=62.31>54.51, 48.42, 46.05) than for the exhaustive CHAID, ANN, and CART algorithms. Conversely, the values of SD ratio=(0.59<0.64, 0.69, 0.71), RMSE=(2.61<2.86, 3.05, 3.12), MAPE (%)=(6.20 <6.84, 7.16, 7.16), RAE=(0.07<0.08, 0.09, 0.09), CV (%)=(7.84<8.62, 9.17, 9.35), and AIC=(293.20<339.77, 358.71, 365.56) were lower for the CHAID algorithm than for the exhaustive CHAID, ANN, and CART algorithms, which indicates better predictive capabilities of the CHAID algorithm.

**Table 2:** Predictive performance of CHAID, Exhaustive CHAID, ANN and CART algorithms.

Algorithm	r	SD ratio	CV (%)	R^2^ (%)	Adj-R^2^ (%)	RAE	RMSE	MAPE (%)	AIC
CHAID	0.80	0.59	7.84	64.48	62.31	0.07	2.61	6.20	293.20
EX.CHAID	0.75	0.64	8.62	57.15	54.51	0.08	2.86	6.84	339.77
ANN	0.71	0.69	9.17	51.69	48.42	0.09	3.05	7.16	358.71
CART	0.70	0.71	9.35	49.28	46.05	0.09	3.12	7.16	365.56

**Figure- 1:**
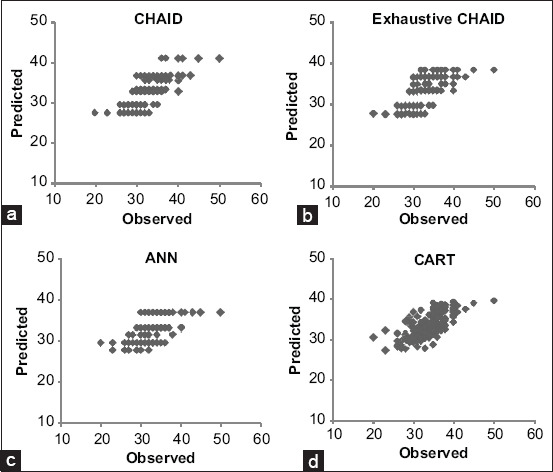
(a-d) Observed *vs*. predicted values of Thalli sheep’s body weight for the prediction models.

We selected the CHAID algorithm as the ideal algorithm according to its values of the goodness-of-fit criteria. In this regard, the CHAID algorithm constructed the tree-based decision tree structure. Figure-2 shows its regression tree diagram. The regression tree structure for the CHAID algorithm had HG, RW, WH, BL, HW, and RL, which were found to be significant independent variables in the live BW prediction in female Thalli sheep. All of the sheep were divided into four subgroups (Node 1, Node 2, Node 3, and Node 4) according to HG (Figure-2). The weight order among Nodes 1-4 was found to be Node 1<Node 2<Node 3<Node 4 (Adjusted p=0.000, F=32.37, df1=3, df2=148), because of the significant differences in BW. Node 1 was the subgroup of sheep with an HG of ≤73.66 cm (BW=29.59 kg). Among all the sheep, Node 2 was the subgroup of sheep with 73.66 cm<HG≤79.75 cm (BW=32.67 kg). The subgroup of sheep with 79.75 cm<HG≤85.09 cm was entered into Node 3 in the decision tree construction of the CHAID algorithm (BW=35.47 kg). Node 4 was the subgroup of sheep with an HG of >85.09 cm (BW=38.23 kg). The sheep incorporated into Node 1 were divided into smaller subgroups (Nodes 5 and 6) in terms of the RW trait. The sheep (RW≤21.59 cm and 73.66 cm≤HG) in Node 5 were lighter in weight than those (with an RW of >21.59 cm and HG of ≤73.66 cm) in Node 6 (Adjusted p=0.015; 27.66 vs. 30.90 kg). Node 2 was further divided into two smaller subgroups (Nodes 7 and 8) according to the WH trait (Adjusted p=0.000, F=22.73, df1=1, df2=50). The average BW of the sheep (WH≤66.04 cm and 73.60 cm<HG≤79.75 cm) in Node 7 was estimated to be 27.60 kg, whereas the average BW of the sheep (66.04 cm<WH and 73.60 cm<HG≤ 79.75 cm) in Node 8 was found to be 33.21 kg. Moreover, Node 3 (the subgroup with 79.75 cm<HG≤85.09 cm) was further divided into smaller subgroups (Nodes 9 and 10) according to BL trait (Adjusted p=0.000, F=22.11, df1=1, df2=44). The sheep with a BL of ≤66.04 cm in Node 9 were lighter in weight than those with a BL of >66.04 cm in Node 10 (Adjusted p=0.000; 29.60 vs. 36.19 kg). Node 4 (the subgroup with 85.09 cm <HG) was further divided into smaller subgroups (Nodes 11 and 12) according to BL trait (Adjusted p=0.045, F=10.02, df1=1, df2=15). The sheep with a BL of ≤73.91 cm in Node 11 were lighter in weight than those with a BL of >73.91 cm in Node 12 (Adjusted p=0.045; 35.66 vs. 41.12 kg). Node 6 (the subgroup with 21.51 cm <RW) was further divided into smaller subgroups (Nodes 13 and 14) in terms of HW trait (Adjusted p=0.011, F=12.22, df1=1, df2=20). The sheep with HW of ≤10.16 cm in Node 13 were lighter in weight than those sheep with HW of >10.16 cm in Node 14 (Adjusted p=0.045; 29.73 vs. 33.42 kg). Node 8 (a subgroup of the sheep with WH of >66.04 cm) was further divided into smaller subgroups (Nodes 15 and 16) according to RL trait (Adjusted p=0.028, F=8.87, df1=1, df2=45). The sheep with RL of ≤15.24 cm in Node 15 were lighter in weight than those sheep with RL of >15.24 cm in Node 16 (Adjusted p=0.028; 32.88 vs. 36.00 kg). Node 10 (a subgroup of the sheep with 66.09 cm <BL) was further divided into smaller subgroups (Nodes 17 and 18) according to BL trait (Adjusted p=0.045, F=8.29, df1=1, df2=39). The sheep with an RL of ≤14.98 cm in Node 17 were lighter in weight than those sheep with an RL of >14.98 cm in Node 18 (Adjusted p=0.045; 33.42 vs. 36.76 kg). Node 0 is the root node. In our analysis, Nodes 1, 2, 3, 4, 6, 8, and 10 were the child nodes, whereas Nodes 5, 7, 9, 11, 12, 13, 14, 15, 16, 17, and 18 were the terminal nodes. The CHAID algorithm declared HG as the most significant predictor of live BW. Some earlier studies also reported that HG is an important predictor for live BW prediction in different goat and sheep breeds [1,24,25].

**Figure- 2:**
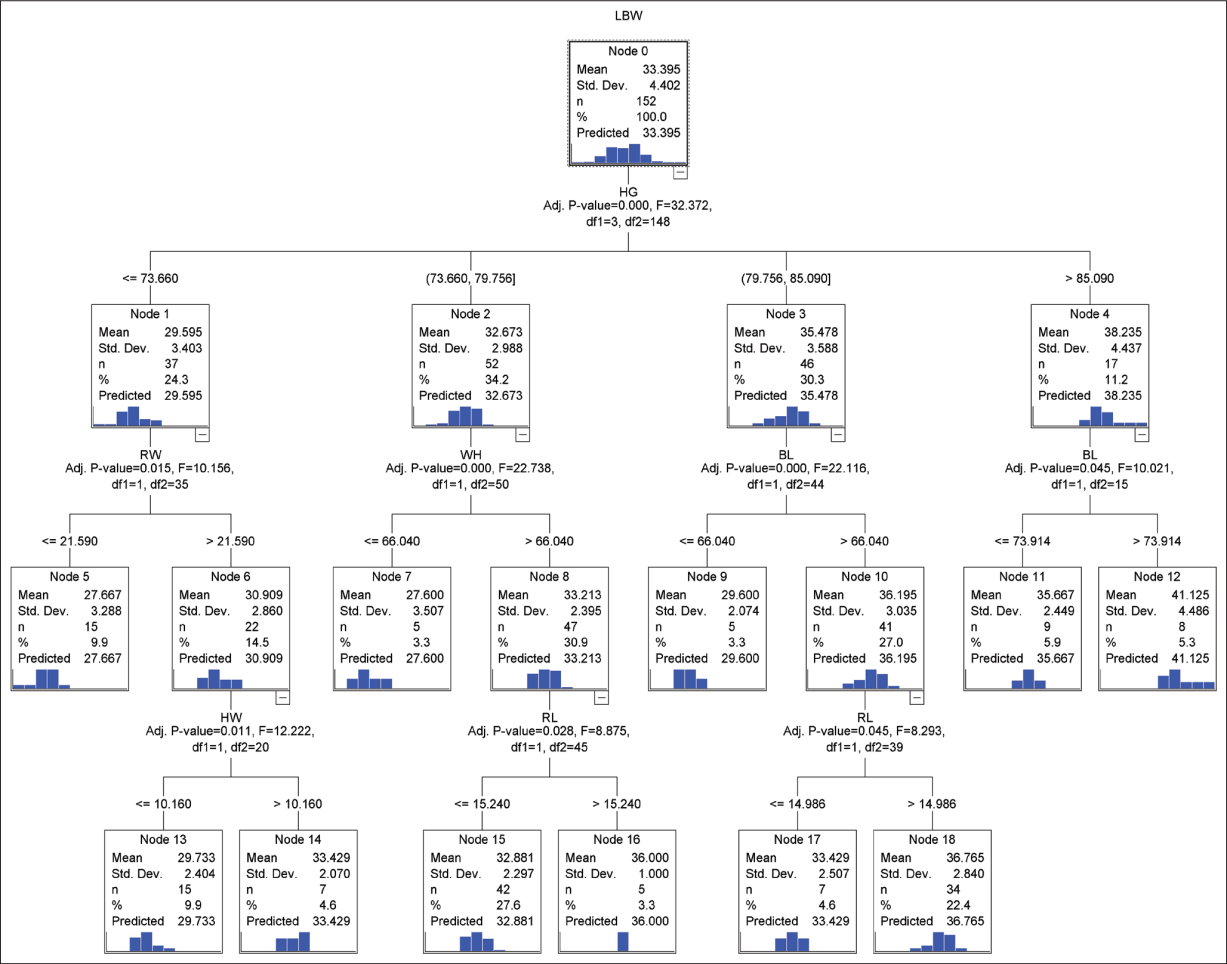
Decision tree constructed for CHAID algorithm.

In general, an assessment of the association between the BW and body measurements obtained using a measuring technique is a meaningful procedure in the BW prediction of sheep. The validity of the procedures heavily relies on the statistical techniques applied by various analysts. In the past studies, the use of data mining algorithms rather than traditional analysis was finitely made in BW prediction through body measurements in sheep [10]. In this study, the predictive performance of the CHAID, exhaustive CHAID, ANN, and CART algorithms used for predicting BW using body measurements in sheep has been evaluated comparatively. The results of the present study results consistent with those of the study by Karabacak *et al*. [11] that included five different breeds of sheep and found that the performance of the CHAID algorithm in terms of BW prediction was better than those of the other algorithms. A study by Ali *et al*. [10] used different data mining algorithms in the prediction of BW in Harnai sheep. They obtained R^2^ (83.77%), correlation coefficients between the observed and predicted BW values (0.91), SD ratio (0.40), CV (5.71%), RMSE (1.50), and RAE (0.05) for the CHAID algorithm. Their estimates were better than the results obtained from the present study. In a similar study conducted by Celik *et al*. [26], they compared the predictive performance of different data mining algorithms in predicting the BW of Mengali rams in Pakistan. They estimated R^2^ (0.90), r (0.94), SD ratio (0.31), and MAPE (6.48) for the CHAID algorithm. The estimates obtained by Celik *et al*. [26] were also better than those in the present study. Compared with the present R^2^ (%) estimated for the CHAID algorithm, Mohammad *et al*. [14] estimated a higher R^2^ (%) value of 72% for the CHAID algorithm in the BW prediction based on WH, chest girth, BL, and breed in indigenous Pakistani sheep. In another study conducted by Khan *et al*. [27], they recorded R^2^=0.84 for the exhaustive CHAID algorithm in the estimation of the BW of Harnai sheep based on significant predictors, such as face length, WH, chest girth, and BL. Their estimates were also better than the results obtained from the present study. The estimation of BW in indigenous Beetal goats in Pakistan according to head girth, neck length, diagonal BL, belly sprung, shank circumference, and rump height input variables was reported by Eyduran *et al*. [16] in the scope of the CART, CHAID, RBF, MLP1, MLP2, and MR modeling. They found better goodness-of-fit criteria (r, AIC, RMSE, SD ratio, and MAD) regarding the CHAID algorithm than those obtained from the present study. The difference in the results may be due to the ecological conditions, breed, rearing systems, wide variation in ages, use of different body measurements and their interface, managerial factors, and statistical tools used in the study. However, it is recommended for further investigators that the predictive performances of the evaluated data mining methods should be used for different sheep breeds and studies with a large number of sheep breeds and efficient factors in the generalization of the results obtained from the present study.

## Conclusion

We found that for the prediction of live BW in Thalli sheep, the CHAID algorithm had a better and more accurate performance than had the exhaustive CHAID, ANN, and CART algorithms because of the higher Pearson correlation coefficient (0.80), R^2^ (64.48%), Adj-R^2^ (62.31%), lower MAPE (6.20%), SD ratio (0.59), RMSE (2.61), RAE (0.07), CV (7.84%), and AIC (293.20). Although all the algorithms can remarkably predict live BW similar to the actual values, the performance of the CHAID algorithm for the live BW prediction through linear body measurements in female Thalli sheep was higher and more precise. Therefore, it is possible to apply the CHAID algorithm for the prediction of actual BW using body measurements. Furthermore, researchers may use these results for comparison purposes and as a reference in future studies.

## Authors’ Contributions

AA, MAU, and AW: Designed the study, collected the data and performed the study. AA: Drafted the manuscript. AA and AW: Analyzed the data. AU: Supervised and helped in interpretation of the results. All authors have read and approved the final manuscript.
